# Molecular dynamics simulations of aggregation and viscosity properties of model asphaltene molecules containing a polycyclic hydrocarbon nucleus with toluene additive under shear interactions†

**DOI:** 10.1039/d3ra06483b

**Published:** 2024-01-15

**Authors:** Baoliang Peng, Lingfang Yuan, Xianqiong Tang, Yao Wang, Yingying Li, Weidong Liu, Yong Pei

**Affiliations:** a Research Institute of Petroleum Exploration & Development (RIPED), PetroChina, Key Laboratory of Oilfield Chemistry, CNPC Beijing 100083 People's Republic of China; b Key Laboratory for Green Organic Synthesis and Application of Hunan Province, Key Laboratory of Environmentally Friendly Chemistry and Applications of Ministry of Education, Xiangtan University Xiangtan 411100 People's Republic of China; c School of Mechanical Engineering and Mechanics, Xiangtan University Xiangtan 411100 People's Republic of China; d PetroChina Liaohe Oilfield Company Panjin 124010 People's Republic of China

## Abstract

Reducing the viscosity of heavy oil is beneficial to the process of oil recovery, so it is of great significance to explore the influence of different factors on the viscosity of heavy oil. In this study, molecular dynamics (MD) simulations were carried out to study the viscosity properties of 15 structurally homologous model polycyclic molecules under shear conditions and with a toluene additive with different concentrations. Over 50 sets of simulation systems were constructed and simulated in this work. The molecular structure effect including the phenyl ring arrangements, alkyl side chain decorations, and heteroatoms, as well as the solvent effect such as the concentration of the toluene additive was comprehensively studied. It was found that under the shear conditions, the more branched the benzene ring in the polycyclic hydrocarbon nucleus, the greater the molecular steric hindrance generated, resulting in higher viscosity compared to O-shaped polycyclic hydrocarbon nucleus molecules. The introduction of alkyl side chains and heteroatoms leads to increased intermolecular interactions and more face-to-face stacking configurations, resulting in an increase in viscosity. However, in comparison, the heteroatoms effect is more pronounced in intermolecular interactions and increases in viscosity. Molecular trajectory analysis further indicates the molecular aggregates undergo continuous fracture and recombination under shear interaction, which is related to the trend of changes in viscosity properties. The current research provides new atomic-level insights into the molecular motion of heavy oil components under shear interaction in the presence of a toluene additive.

## Abstract 2

Molecular dynamics (MD) simulations were carried out to study the aggregation behavior and viscosity properties of 15 structurally homologous model asphaltene molecules under shear conditions and with a toluene additive with different concentrations.

## Graphical abstract

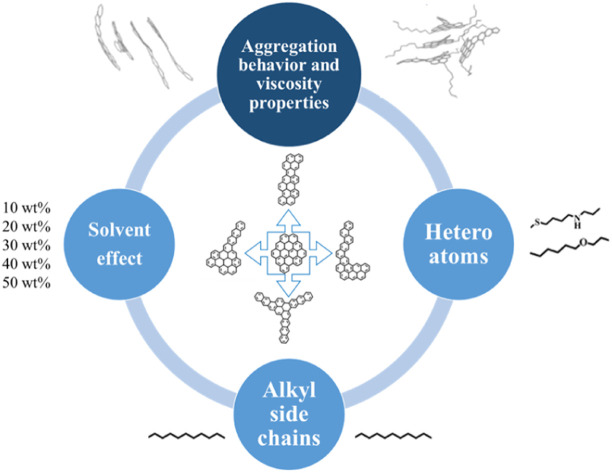

## 1. Introduction

How to improve the utilization efficiency of heavy crude oil in the context of the continuous reduction of conventional crude oil is an important issue that the petroleum industry is highly concerned about. A major difficulty in producing and transporting the heavy crude oil is because of its high viscosity. Asphaltene is the main contributor to high viscosity of heavy oil. The problem of self-aggregation and precipitation caused by asphaltene components in heavy crude oil has always plagued the exploration, extraction, storage, transportation, and processing processes of the petroleum industry.^1–3^ Therefore, studying the viscosity reduction of asphaltene can help improve the utilization efficiency of crude oil.^4–7^

Asphaltene is composed of condensed polyaromatic rings, alkane chains, and heteroatoms such as nitrogen, oxygen, and sulfur atoms, *etc.*^8–10^ While the majority of asphaltenes may have a common architectural structure containing a polycyclic aromatic core and peripheral aliphatic chains, their size and aromaticity vary considerably. Due to intermolecular interactions such as the π–π interaction, asphaltenes tend to self-associate forming nanoaggregates, and then nanoaggregates will further form the coagulate, which eventually leads to flocculation.^11–14^ Some studies have shown that asphaltene aggregation is influenced by many factors such as the molecular structure and concentration of asphaltenes, temperature and pressure, and solvent types.^15,16^ Among these factors, the molecular structures are of particular interest due to the great diversity; for example, the number and position of aromatic ring structures, polarity, alkyl side chains, molecular weight, and molecular symmetry, *etc.*

In recent years many kinds of viscosity-reducing agents have been investigated, such as light oil, low molecular multifunctional molecules, alcohols, *etc.* Recent experimental work indicated that many of these additives do not affect asphaltene association. Most of the additives showed that only dilution affected the asphaltene viscosity. Hasan *et al.* studied the viscosity behavior of heavy crude oil when it is blended with alcohol of 10% and 20% by volume.^17^ The presence of 10% alcohol causes viscosity reduction by almost 80% at 25 °C. Further addition of addition of alcohol can cause more viscosity reduction. They attribute these effects to the interactions between the hydroxyl functions and some functionalities of the asphaltenes. The blending of heavy crude oil with ethanol enhances the flowability of the heavy crude oil. Mortazavi-Manesh and Shaw *et al.* investigated the effect of diluents (*n*-heptane, toluene, and toluene : butanone (50 : 50 vol%)) on the non-Newtonian behavior of Maya crude oil including shear thinning and thixotropy at temperatures from 258 to 333 K.^18^ They concluded that toluene : butanone (50 : 50 vol%) is more effective in decreasing oil viscosity than two other diluents tested.

Despite a lot of work having been done experimentally, the atomic-level details of the molecular aggregation behaviors of the heavy oil component under the interaction of viscosity-reducing agents were largely unknown, which cannot be directly observed experimentally. Notably, in the past few decades, experimental and theoretical work has made great efforts to understand the aggregation behavior of asphaltene molecules. In experimental terms, some instrumental characterization methods such as small angle neuron scattering (SANS), small angle X-ray scattering (SAXS), wide-angle X-ray scattering (WAXS), Rayleigh scattering, nanofiltration and dynamic light scattering (DLS)/photon correlation spectroscopy (PCS) were used to study the aggregation behavior of asphaltenes.^19–21^ It was found that the size of the asphaltene aggregates depends to a large extent on the structure and composition of the asphaltenes. Moreover, the skewed parallel stacking of polycyclic nuclei within the asphaltene nanoaggregates are commonly proposed.^22–28^ On the theoretical simulation aspect, the atomistic molecular simulations have been extensively carried out to study the interactions between asphaltene molecules and their molecular aggregate structures. The molecular structure effect, and the effect of salt ions, solvent molecules such as toluene and heptane, and temperatures on the asphaltene molecule aggregation behaviors were extensively explored by molecular dynamic simulations.^29–37^ Very recently, the machine-learning approach was introduced to identify a reduced set of model molecules representative of the diversity of asphaltene by Pétuya *et al.*^38^ The studies highlighted the complex and diverse effects of molecular polydispersity on the aggregation process of asphaltene. Their simulation results indicate that when studying the aggregation process of asphaltene, it is necessary to consider molecular polydispersity. The study reported by Javanbakht *et al.* also demonstrates the importance of polydispersity on asphaltene aggregation and provides a lower limit of approximately 375 molecules in such a mixture to represent the two stages of aggregation.^39^

On the other hand, the dynamics of asphaltene molecules under shear field has also attracted interest in the research community. It was found that increasing shear force can enhance the aggregation rate of asphaltene, and the average steady-state flocculent size of asphaltene decreases with increasing shear force. Simultaneously increasing the concentration of asphaltene in the solution or reducing the ratio of toluene to heptane can increase the growth rate of flocs and the size of steady-state flocs.^40–43^ Bahrami *et al.* conducted experiments on the effect of shear rate on the aggregation of asphaltene in heptane toluene mixtures at constant temperature and pressure, and the results showed that under the action of shear force, the aggregated particles formed by asphaltene were denser and the formation time was shortened.^44^ Song *et al.* used dissipative particle dynamics to study the effect of shear field rate on the dispersion behaviors of asphaltene molecules in heptane. The simulation results showed that asphaltene molecules mostly stack face-to-face and T-shaped, and shear field can damage the stacking of asphaltene molecules to varying degrees. The effect of shear force on archipelagic asphaltene molecules is higher than that of continental asphaltene molecules, mainly due to the stretching of archipelagic asphaltene molecules by the shear field.^45^ Nassar *et al.* believe that the decrease in viscosity of asphaltene in the shear field is due to the disruption of the bonding force between asphaltene molecules in the shear field, resulting in an increase in the dimer formation free energy.^46^

In this study, the effects of toluene additive on the viscosity of model asphaltene molecules containing polycyclic cores in the presence of shearing field were investigated by molecular dynamics simulations. The main purpose of this study is to investigate how viscosity reduction additive (toluene) affect the viscosity and molecular interactions of asphaltene molecules with different topological structures. To achieve this goal, the dispersion behavior and viscosity properties of five structurally homologous “continental” model alphaltene molecules with different benzene ring arrangements, alkyl side chains, and heteroatoms was simulated using non equilibrium molecular dynamics with different toluene additive concentrations. Over 50 sets of simulation systems were constructed and each system was simulated over 60 ns. The atomic level insights of the molecular structure effect of asphaltene, the effect of toluene additive concentrations on the aggregation and dispersion behaviors and shearing viscosity properties of these model asphaltene were obtained.

## 2. Molecular simulation method and models

### 2.1 Construction of molecular models

**Fig. 1:**
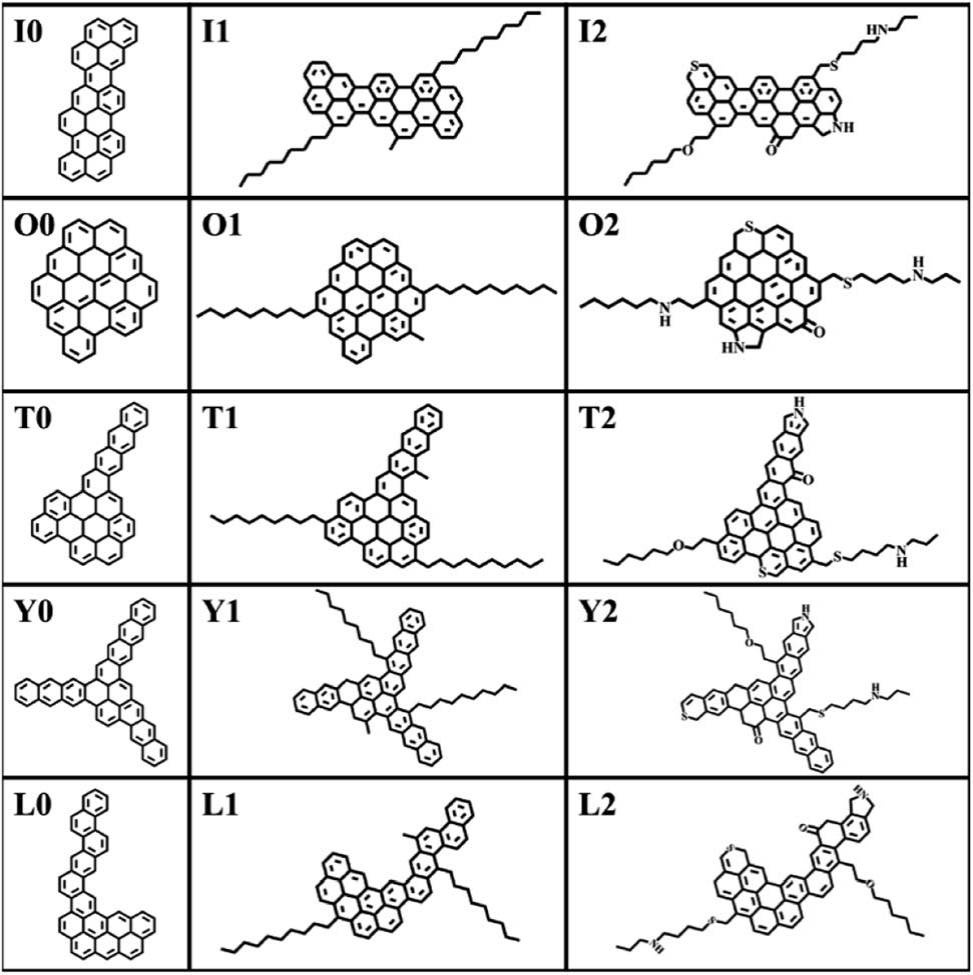
Molecular structure models of 15 model asphaltene molecules.

The construction of the structural model of asphaltene compounds is based on the structure of coal asphaltene molecules measured by Schuler *et al.* using atomic force microscopy.^8,14^ We selected five representative condensed rings with different benzene ring arrangements, namely O, I, T, Y, and L, as the nucleus, and then further introduced alkyl side chains and hetero atoms to construct 15 asphaltene molecular models. According to their molecular structure and composition, these molecular models can be divided into three categories: the PAHs-I0, PAHs-O0, PAHs-T0, PAHs-Y0, and PAHs-L0; the PAHs-I1, PAHs-O1, PAHs-T1, PAHs-Y1, and PAHs-L1 containing alkyl side chains, and the PAHs-I2, PAHs-O2, PAHs-T2, PAHs-Y2, and PAHs-L2 containing hetero atoms. All molecular structures are shown in Fig. 1. In recent theoretical studies by Law *et al.*,^47^ the effect of asphaltene molecular polydispersity was emphasized to properly simulate the properties such as aggregation behaviors of asphaltene systems. In the present study, we focused on the effect of local molecular structure effects such as aromatic core stacking pattern, alkyl side chains and heteroatom on the inter-molecular interactions, viscosity reduction and molecular dispersion behaviors upon the addition of solvent molecules under shear conditions. So, the five types of structurally homologous model alphaltene molecules with different benzene ring arrangements, alkyl side chains, and hetero atoms were used.

To determine the relative position and orientation of asphaltene molecules, based on the flat structure of asphaltene molecules, the centroid (COM) of asphaltene molecules is first calculated. Then, the peak of the radial distribution function (RDF) is used to determine the distance distribution between the COM of the asphaltene. The COM distance of asphaltene molecules stacked face-to-face is the smallest, corresponding to the first peak of RDF. The COM distance of offset stacked asphaltene molecules is greater than that of face-to-face stacked asphaltene molecules, which corresponds to the position of the second peak of RDF. The COM of the “T-shaped” stacked asphaltene molecules is defined as the position of the third peak of RDF. The average distance (AD) between aggregate molecules also can be obtained through the radial distribution function. The AD is used to analyze the size and quantity of alphaltene molecular aggregates in the simulation box.

### 2.2 Molecular dynamics simulations

The simulation process of all asphaltene systems includes three steps: (I) in the initial simulation box, 40 asphaltene molecules of the same type are randomly distributed, and the initial density of the system is set to 0.6 g cm^−3^, which is lower than the density of conventional asphaltene. (II) Calibration of density and energy of randomly generated simulation boxes (including detection of kinetic and potential energy). Each system was balanced through a 20 ns isobaric isothermal (NPT) ensemble simulation at a constant pressure of 1 bar, with a temperature maintained at 300 K. This gives the system an appropriate density and box size. (III) Perform 60 ns NVT simulation using a Nosé-Hoover thermostat at 300 K. In this stage of simulation, we applied a shear field with a shear rate of 1 × 10^−7^ fs^−1^ to the simulation box, with a time step of 1 fs. (IV) The data collection stage after balancing. The atomic position, force, velocity, potential energy generated after the complete relaxation and equilibrium process (after 10 ns NVT simulation) of the system were collected for subsequent analysis.

All MD simulations are executed using the Large-scale Atomic/Molecular Massively Parallel Simulator (LAMMPS) package.^48^ The force field parameter selection is PCFF force field. PCFF is a fully atomic force field that can provide good accuracy in liquid properties (density and cohesive energy) and molecular conformation.^49^ PCFF mainly consists of bonding and non-bonding potential energy terms, while non-bonding terms consist of long-range electrostatic interactions and short-range van der Waals (vdW) interactions, can accurately predict the structure and thermodynamic properties of petroleum components.^50–53^ The Lennard-Jones potential describes the non-bonding interactions between these sites, with a cutoff value of 12.0 Å. The long-range Coulomb interaction is processed using the Particle Particle Particle Mesh (PPPM) algorithm,^54^ with a convergence parameter of 10^−4^. The time step and time interval for collecting data are set to 1 fs and 1 ps, respectively. The temperature was set 300 K and the pressure was 1 bar in the simulation. In each simulation, 40 specific types of model asphaltene molecules and different numbers of toluene molecules are randomly placed in a cubic box. Periodic boundary conditions apply in all directions. The VMD software^55^ was used for trajectory analysis and visualization. The velocity verlet algorithm is used for integration in MD simulation.

**Fig. 2:**
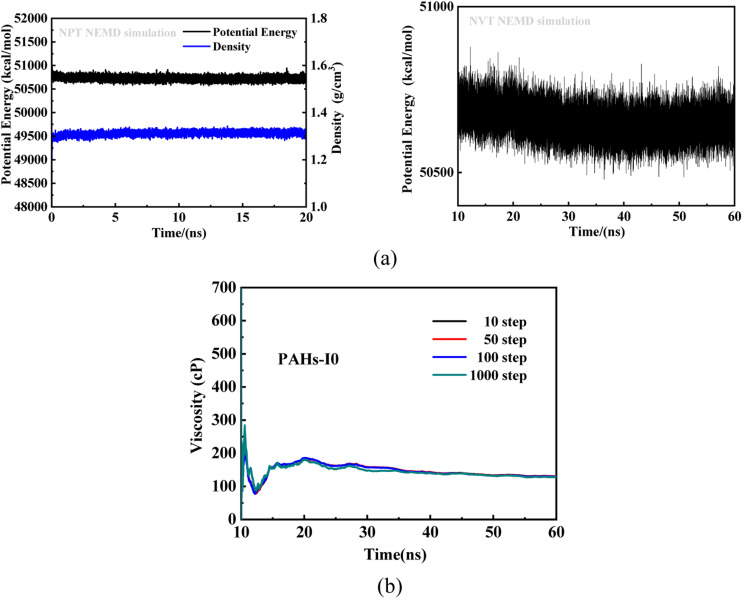
(a) Potential and density evolution curves of PAHs-I0 under the NPT and NVT simulations at condition of the toluene mass concentration being 10 wt%. (b) The convergence of the viscosity of PAHs-I0 in the 60 ns NVT simulations.

To investigate the effect of toluene additive concentration on the aggregation and viscosity properties of asphaltene, we investigated the shear viscosity properties of asphaltene molecules with different structures under different toluene solvent concentrations. This work conducted molecular simulation studies on a total of 50 model systems (Table 1) with different toluene additive concentrations. As shown in Fig. 2a, taking the NPT and NVT potential energy convergence diagram of PAHs-I0 system under the condition of toluene mass concentration of 10% as examples, it can be seen that the density and potential energy of all simulation systems have fully converged. For the calculation of viscosity, as shown in Fig. 2b, within a total simulation time of 60 ns, the trajectory of the first 10 ns shows significant viscosity fluctuations, this is not included in the trajectory analysis. Only the simulated trajectory of 50 ns after simulation is recorded and analyzed. At the same time, we compared the viscosity data obtained by averaging every 10, 50, 100, and 1000 steps, and found that the viscosity obtained by the four ways of recording data was completely convergent and the convergence values were almost consistent.

**Table 1:** A total of 50 model systems with different toluene additive concentrations

Type	Tol/00 wt%	Tol/10 wt%	Tol/20 wt%	Tol/30 wt%	Tol/40 wt%	Tol/50 wt%
**PAHs-0**						
O	✓	✓	✓	✓	✓	✓
I	✓	✓	✓	✓	✓	✓
T	✓	✓	✓	✓	✓	✓
Y	✓	✓	✓	✓	✓	✓
L	✓	✓	✓	✓	✓	✓

**PAHs-1**						
O	✓	✓	✓	✓	✓	✓
I	—	—	✓	—	—	—
T	—	—	✓	—	—	—
Y	—	—	✓	—	—	—
L	—	—	✓	—	—	—

**PAHs-2**						
O	✓	✓	✓	✓	✓	✓
I	—	—	✓	—	—	—
T	—	—	✓	—	—	—
Y	—	—	✓	—	—	—
L	—	—	✓	—	—	—

## 3. Results and discussions

### 3.1 Effect of distribution of phenyl rings on the aggregation and viscosity of asphaltene

**Fig. 3:**
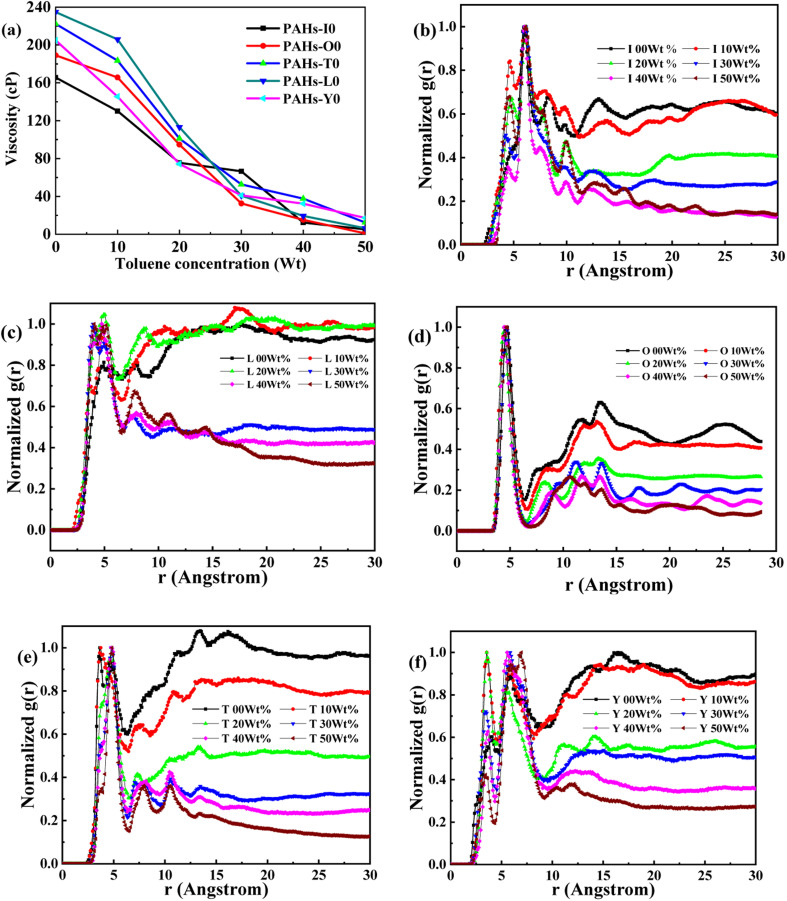
(a) The viscosity of PAHs-0 series asphaltene molecules under different toluene additive concentration. (b–f) The NRDF curves of PAHs-0 series alphaltene molecules.

Asphaltene is a key factor in the high viscosity of heavy oil. This section takes five molecules of the PAHs-0 series (PAHs-I0, PAHs-O0, PAHs-T0, PAHs-Y0, PAHs-L0) as examples to study the effect of the distribution pattern of benzene rings in the continental type asphaltene molecules on their viscosity properties. As shown in Fig. 3, the simulation results show that there are significant differences in the viscosity values of asphaltene molecules with different benzene ring arrangements, indicating a benzene ring distribution pattern significantly affect the molecular viscosity property. At the same time, it can also be seen that when toluene solvent is added to the asphaltene molecular system, toluene molecules will destroy the aggregate structure of the asphaltene, leading to a decrease in viscosity.

**Fig. 4:**
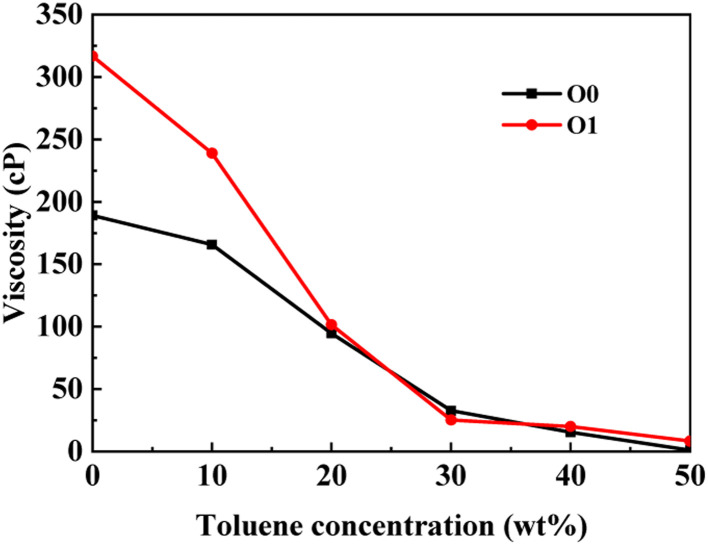
Comparison of the viscosity of PAHs-0 and PAHs-1type asphaltene molecules.

When the concentration of toluene solvent is 0 wt%, the maximum viscosity difference of the five asphaltene molecules is 69.2 cP. When the concentration of toluene solvent is 10 wt%, the viscosity difference increases to 75.7 cP. However, when the concentration of toluene increases to 20 wt%, the viscosity difference between them decreased to 38.8 cP. As the concentration of toluene solvent further increases, the viscosity difference between different configurations of asphaltene molecular systems is no longer change significantly, and the benzene ring distribution effect of asphaltene molecules is almost not seen at this time. At the same time, it can be observed that when the concentration of toluene solvent is 20 wt%, the viscosity of the five asphaltene systems decreases to a relatively stable point, indicating that asphaltene can reach the optimal dissolution state in 20 wt% toluene. It is worth noting that when the concentration of toluene solvent is in the range of 0 wt%–20 wt%, the viscosity order of the five asphaltenes is: PAHs-L0 > PAHs-T0 > PAHs-O0 > PAHs-I0 > PAHs-Y0. This result deviates from traditional views, as the current simulation results show that the viscosity of continental asphaltene with an O-type structure is lower than that of L-type and T-type asphaltene molecules. To understand this trend, we conducted Normalized Radial Distribution Function (NRDF) analysis on the aggregate structures formed by five asphaltene molecules in the PAHs-0 system. The NRDF is defined 
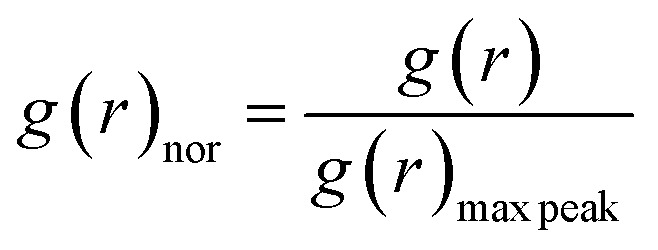
, where *g*(*r*)_max_ is the maximum peak of the radial distribution function (*g*(*r*)).^54^ The closer the convergence value of NRDF is to 1, the more disorderly the intermolecular stacking becomes. The closer the convergence value of NRDF is to 0, the more orderly the intermolecular stacking becomes.^56–59^ As shown in Fig. 3b–f, when the concentration of toluene solvent is below 20 wt%, the converged NRDF for almost all asphaltenes is >0.5, indicating that the stacking of asphaltene molecules is relatively disordered. The order of NRDF is PAHs-L0 > PAHs-Y0 > PAHs-T0 > PAHs-I0 > PAHs-O0. The results show that PAHs-O0 type asphaltene molecules have a relatively ordered molecular stacking structure, which corresponds to the larger planar configuration of such molecules. However, the viscosity of O-type asphaltene is inconsistent with the trend of the normalized radial distribution function. By further analyzing the viscosity curve of the five PAHs-0 systems, it can be found that the slope of the viscosity decrease curve for the O-type asphaltene is the smallest with the increase of toluene solvent, while the slope of the viscosity decrease curve for Y-type asphaltene is the largest with the increase of toluene solvent. From this, it can be seen that toluene solvent has a significant impact on the viscosity properties of asphaltene molecules containing a large number of benzene rings in branching distribution pattern. As shown in Fig. 4, PAHs-O0 is a typical continental asphaltene molecule with multi benzene ring fusing into a big-ring configuration, and its molecular movement is relatively smooth under shear. The distribution of benzene rings in the PAHs-Y0 is highly branched, and the movement of the molecule under shear exhibits significant steric hindrance. This phenomenon indicates that the distribution of phenyl rings in the asphaltene molecules affected the aggregation behavior and viscosity properties under external shear stress. The branching distributed benzene rings mainly causes molecular structural hindrance effect, which leads to an increase in viscosity under shear conditions.

### 3.2 Branch chain effect

This section conducted systematic molecular dynamics simulations on five types of asphaltene molecules belong to the PAHs-1 series to explore the effect of alkyl branching on the viscosity of asphaltene. The toluene additive effect is first analyzed. As shown in Fig. 4, when the concentration of toluene solvent is below 20 wt%, the viscosity of PAHs-O1 asphaltene molecules is almost twice that of PAHs-O0 asphaltene molecules. When the toluene solvent exceeds 20 wt%, the viscosity difference between PAHs-O0 and PAHs-O1 rapidly decreases and tends to be consistent, indicating that the effect of side chains on the viscosity of asphaltene molecules can be almost ignored when the solvent exceeds 20 wt%. When the concentration of toluene solvent is below 20 wt%, the alkyl chains increase the molecular structure hindrance of asphaltene, leading to an increase in the viscosity of asphaltene.

**Fig. 5:**
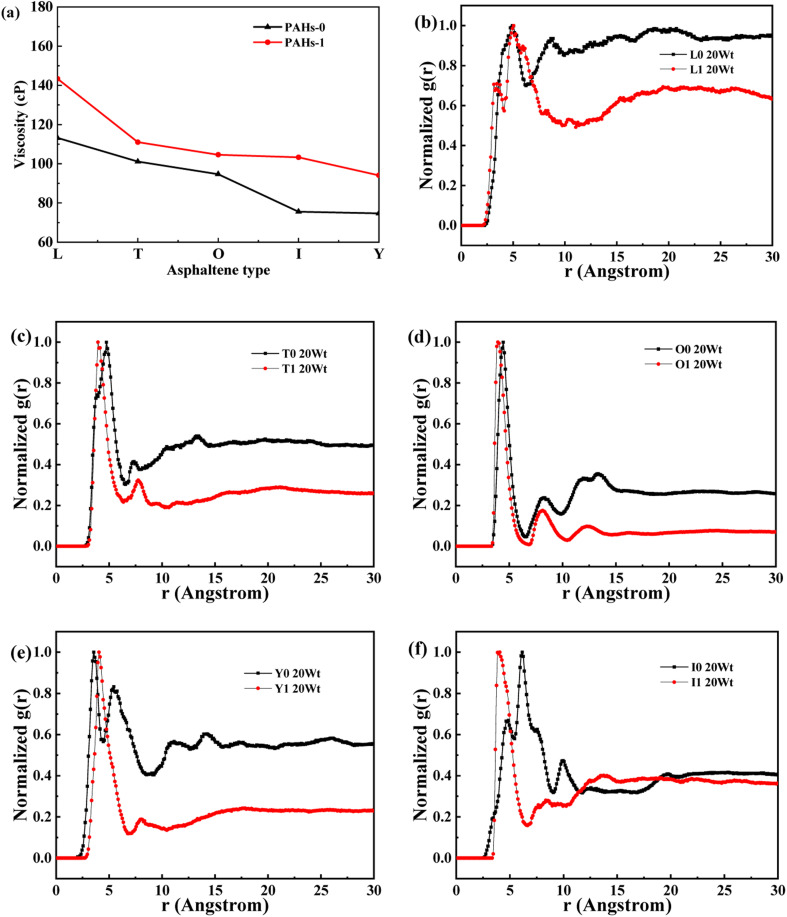
(a) Comparison of the viscosity of PAHs-0 and PAHs-1 asphaltene molecules. (b–f) The NRDF of PAHs-0 and PAHs-1 series asphaltene molecules at 20 wt%.

In order to further understand the effect of alkyl chains on the aggregation mode and the viscosity, the normalized radial distribution function and molecular stacking configuration of PAHs-1 type asphaltene molecules with toluene additive concentration at 20 wt% were analyzed. By comparing the PAHs-0 type and PAHs-1 type asphaltene molecules, as shown in Fig. 5, at a concentration of 20 wt% toluene additive, the largest viscosity difference between PAHs-L0 and PAHs-L1 series asphaltene molecules is 30.2 cP, the largest viscosity difference between PAHs-I0 and PAHs-I1 series molecules is 27.8 cP, the largest viscosity difference between PAHs-Y0 and PAHs-Y1 series molecules is 19.4 cP, and the largest viscosity difference between PAHs-O and PAHs-T series molecules are 9.9 cP. These results show that the same alkyl chains have different effects on asphaltene molecules with different polyaromatic nuclei structures. As shown in Fig. 5b–f, except for the Y-type molecules, the maximum peaks of NRDFs of PAHs-1 molecules shifted to smaller value comparing to that of PAHs-0 molecules. This indicates that the presence of alkyl side chains reduces the stacking distance between asphaltene molecules. In addition, the convergence values of the NRDF of the PAHs-1 series are lower than those of PAHs-0 series. This indicates that branching alkyl chains increase the interaction between asphaltene molecules, making them more inclined towards orderly face-to-face stacking.

**Fig. 6:**
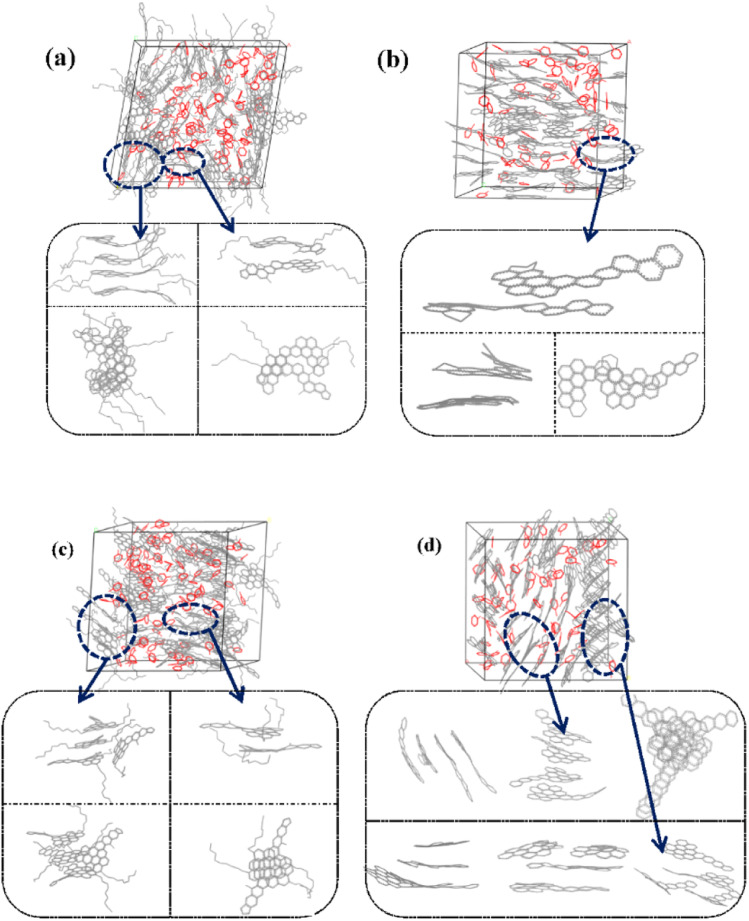
Snapshot structures of (a) PAHs-L1, (b) PAHs-L0, (c) PAHs-T1, and (d) PAHs-T0 type asphaltene molecular aggregates in the presence of 20 wt% toluene additive. Red lines shows the toluene molecules, grey lines represent the carbon atom skeletons. H atoms are omitted.

Fig. 6 and S1† showed the stacking configuration of asphaltene molecules in equilibrium state. It can be observed that toluene molecules have not entered the interior of the face-to-face stacking structure of asphaltene molecules, and only exist in the interstices between aggregates. Fig. 6a and b display typical packing structure snapshots of PAHs-L type asphaltene molecules, it can be seen that PAHs-L0 type asphaltene molecules exhibit planar stacking. However, due to the L-shaped distribution of benzene rings, they did not stack perfectly to form a face-to-face configuration, but instead showed offset π-stacking, as shown in the structural motifs in the enlarged image. In contrast, the polyaromatic core of PAHs-L1 type asphaltene molecules with alkyl branched chains exhibit more perfect face-to-face stacking, including head-to-head stacking and head-to-tail stacking. The similar side-chain effect was also seen in other systems. As shown in Fig. 6c and d, for PAHs-T0 type asphaltene molecules, the central part of polyaromatic cores form face-to-face stacking conformation, while the other branched benzene rings exhibit random arrangement. For PAHs-T1 type asphaltene molecules containing side chains, they have the same dense ring core face-to-face stacking structure, but alkyl side chains hinder the movement of PAHs-T1 type asphaltene molecules, resulting in more face-to-face stacking configurations. The snapshots of PAHs-I type asphaltene molecules are shown in Fig. S1,† where the presence of branched chains enhances the interaction between PAHs-I type asphaltene molecules. PAHs-I1 type asphaltene molecules exhibit typical face-to-face stacking. For the PAHs-Y type asphaltene, as shown in Fig. S1,† due to the highly branched benzene ring in PAHs-Y0 asphaltene molecules, a poor stacking order is formed. Under the action of alkyl branched chains, the interaction between PAHs-Y1 type asphaltene molecules is enhanced, and the face-to-face stacking configuration increases obviously. The snapshot of PAHs-O type asphaltene molecules exhibits a unique stacking conformation. As shown in Fig. S1,† PAHs-O0 type asphaltene molecules form a long-range face-to-face stacking structure. In contrast, PAHs-O1 type asphaltene molecules cannot form long-range face-to-face stacking due to steric hindrances of branched chains, but the distance between asphaltene molecules is shortened. Based on the above results, the branching alkyl chains enhanced the interactions between PAHs-1 type asphaltene molecules. The face-to-face stacking configuration is increased for most PAHs-1 type molecules.

### 3.3 Heteroatoms effect

**Fig. 7:**
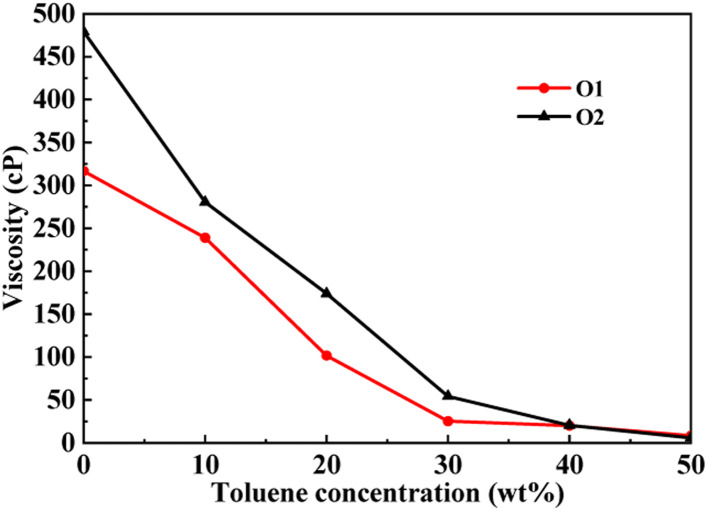
A comparison of the viscosity of PAHs-O1 and PAHs-O2 asphaltene molecules at different toluene concentrations.

This section conducted molecular dynamics simulations on the PAHs-2 system to investigate the effect of hetero atoms on the aggregation behavior and viscosity of asphaltene molecules. As shown in Fig. 7, during the process of increasing the toluene additive to 10 wt%, the slope of viscosity decrease of PAHs-O2 asphaltene is greater than that of PAHs-O1 asphaltene. When the concentration of toluene additive is between 10 wt%–30 wt%, the viscosity decrease rate of PAHs-O1 asphaltene and PAHs-O2 asphaltene is similar. When the concentration of solvent exceeds 40 wt%, the two systems show the similar viscosity value. Therefore, the hetero atoms effect is explored through comparison of the viscosity, normalized radial distribution function, and trajectory of PAHs-1 system and PAHs-2 system with 20 wt% toluene additive.

**Fig. 8:**
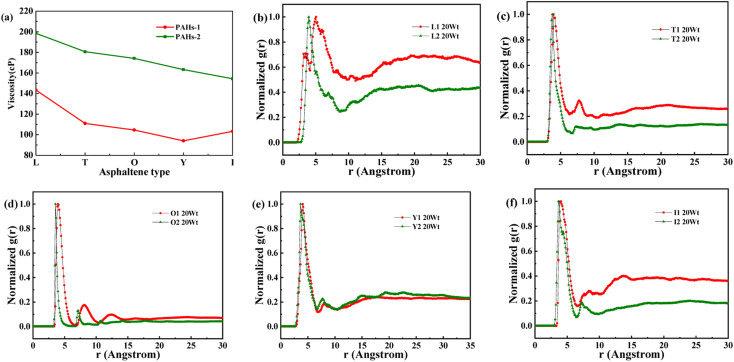
(a) A comparison of the viscosity of PAHs-1 and PAHs-2 type asphaltene molecules at 20 wt% toluene concentration. (b–f) NADF curves of different PAHs-1 and PAHs-2 type asphaltene molecules.

As shown in Fig. 8a, when the concentration of toluene additive is 20 wt%, the viscosity difference between PAHs-1 and PAHs-2 ranges from 51.1 cP to 69.6 cP. For comparison, the viscosity difference between PAHs-0 and PAHs-1 asphaltene molecules is only 9.9 cP to 27.8 cP. This shows that the introduction of hetero atoms has a much greater impact on the viscosity of asphaltene molecules than the influence of branched chains on viscosity. Analyzing all NRDFs, as shown in Fig. 8b–f, the positions of the maximum peaks of NRDFs for asphaltene molecules containing hetero atoms have shift to smaller values comparing with the PAHs-1 asphaltene molecules, indicating that the introduction of hetero atoms further narrows the distance between asphaltene molecules, resulting in more face-to-face stacking. To verify that the hetero atom effect is the key reason of viscosity increase, the molecular polarity is analyzed. The dipole moments were calculated for all asphaltene molecules using the Gaussian 09 package^60^ at the B3LYP/6-31g* theoretical level and the PCFF force field parameters (Table S1†). It can be seen that the dipole moment of the molecules in the PAH-2 series have the largest dipole moment due to introduction of hetero atoms. These results confirms that hetero atom effect is a key factor for the increase in viscosity of asphaltene. The molecular interactions were enhanced by the larger dipole moments caused by the introduction of hetero N, S atoms. We note that this finding agrees well with previous studies by Santos Silva *et al.*,^61^ which indicated the heteroatom substitution on the conjugated core do not modify the shape of the nanoaggregate but change considerably the energy of interaction between asphaltene molecules.

The simulation snapshot of the PAHs-2 system after NVT ensemble calculation is shown in Fig. S2.† It can be seen that the toluene molecules did not enter the interior of the face-to-face stacking of asphaltene molecules, only existing in the interstices of the aggregates. Comparing the snapshots of the PAHs-2 and PAHs-1 systems, it can be observed that the asphaltene molecules in the PAHs-2 system formed more perfect face-to-face stack configuration. It is particularly noteworthy that even the PAHs-Y2 type asphaltene molecules exhibit many face-to-face stacking configurations, indicating the hetero atoms can enhance the aggregation of asphaltene, but their effects on asphaltene molecules with different structures are different.

### 3.4 Fracture recombination effect of asphaltene aggregates

**Fig. 9:**
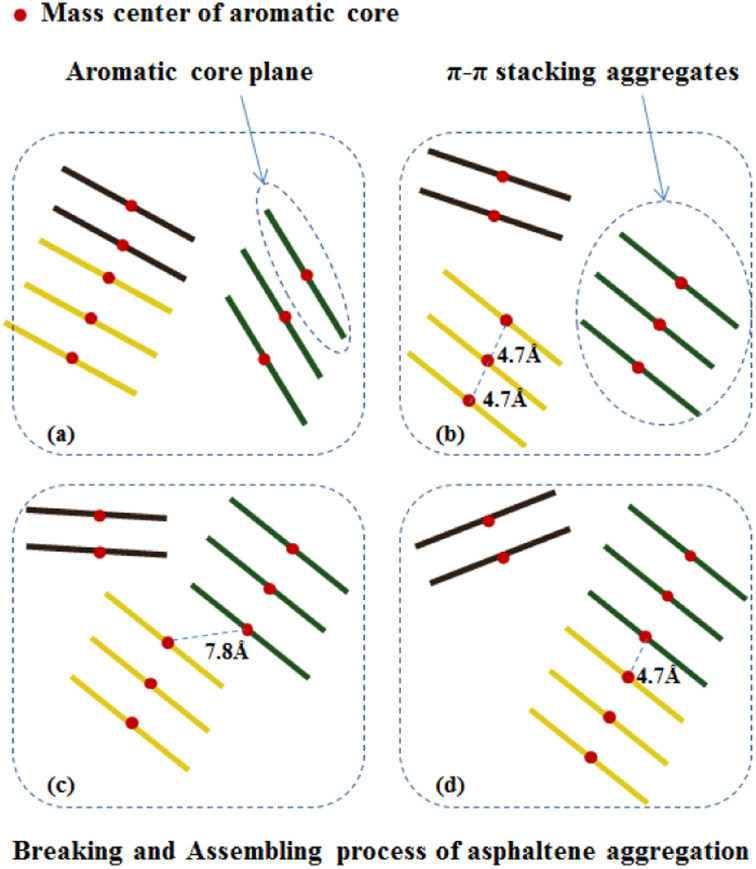
Schematic illustration of the process of fracture and recombination of asphaltene molecular aggregate under shear interactions: (a) contains two aggregates, (b) two aggregates break into three aggregates, (c) the yellow aggregates gradually move to the right, and in (d) the distance between the yellow aggregates and the other aggregates less than 4.7 Å, so the two aggregates merge into a larger one. The quantitative criterion is that the distance between the centers of mass of the two asphaltene molecules ≤4.7 Å.

Under the action of shear fields, the aggregates of asphaltene molecules exhibited fracture-recombination behaviors. By investigating the MD trajectory of asphaltene molecules in the box within 50 ns, it can be clearly observed that under the combined action of shear field and toluene additive, the aggregates of asphaltene molecules exhibit continuous aggregation and fragmentation processes. Fig. 9 schematically illustrates the typical process of fragmentation and recombination of asphaltene molecular aggregates, which includes the formation of smaller molecular aggregates under shear and toluene interactions, and the mutual attraction and recombination of smaller molecular aggregates to form new aggregates.

The aggregation of asphaltene molecules can be quantified based on their distance as a standard. Currently, three distance standards are commonly used: (1) the distance between the closest atoms on two adjacent molecules; (2) the distance between an atom in two adjacent molecules; and (3) the distance between the centroids (COM) of two adjacent molecules. The first or third criterion is used the most. In a recent study carried out by Ghamartale *et al.*,^62^ the distance between the closest atoms was used and the z-averaged aggregation numbers, *g*_z_, was used to calculate the aggregate size. The authors also discussed the suitable criteria that predict the aggregates for a certain type of molecules. In this study, we used the distance between the centroids (COM) of the aromatic cores of two adjacent molecules and applied a cutoff threshold of 0.47 nm as the standard for asphaltene aggregation, which was previously used to counter the nanoaggregate of model asphaltene molecules.^63^

**Fig. 10:**
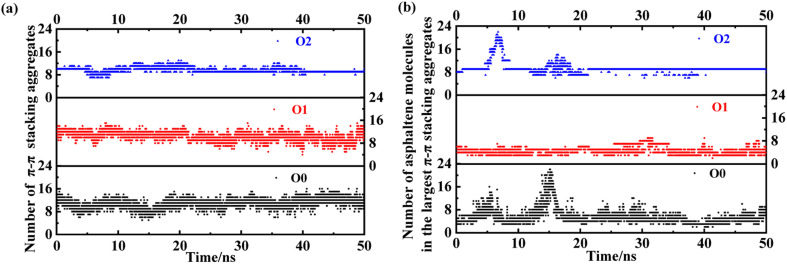
Evolution of the (a) number of π–π stacking aggregate and (b) size of the largest face-to-face stacked PAHs-O0, PAHs-O1, and PAHs-O2 asphaltene molecular aggregate with 20 wt% toluene additive.

Here, we take the PAHs-O series of asphaltene molecules as an example to analyze the number and size changes of asphaltene molecular aggregates under shear under the condition of 20 wt% toluene. As shown in Fig. 10, comparing PAHs-O0, PAHs-O1, and PAHs-O2, the analysis results show that the stability of the number of asphaltene aggregates in the box is relative. Throughout the simulation process, the number of aggregates in PAHs-O0, PAHs-O1, and PAHs-O2 has been constantly changing. Among them, the number of aggregates of PAHs-O0 asphaltene molecules fluctuates the most significantly, and the size change of the largest aggregate is significant. The number of aggregates of PAHs-O1 asphaltene molecules remains within a relatively stable range, and the presence of alkyl branched chains significantly leads to a decrease in the size of asphaltene aggregates. The number of aggregates of PAHs-O2 asphaltene molecules is relatively stable, and the size of the largest asphaltene aggregate is also the most stable. This indicates that alkyl branched chains weaken the intermolecular motion of asphaltene, while polar molecules further weaken the intermolecular motion of asphaltene. This may also be the reason why the viscosity of PAHs-O2 > PAHs-O1 > PAHs-O0. At the same time, in the presence of alkyl chains and hetero atoms, the maximum aggregate size of asphaltene will further increase, which is the most direct evidence of hetero atoms promoting the self-aggregation behavior of asphaltene in the shear field.

## 4. Conclusion

The viscosity properties of 15 asphaltene molecules containing the homologous fused benzene rings under the action of shear fields at a shear rate of 1 × 10^−7^ fs^−1^ to the simulation box were studied using the molecular dynamics simulations. The simulation results indicate the benzene ring distribution in the polycyclic core has a great impact on the viscosity of asphaltene molecules. The viscosity of L-type and T-type asphaltene molecules is higher than that of O-type and Y-type asphaltene molecules due to the significant steric effect of branched distributed benzene rings. The introduction of alkyl branched chains and hetero atoms enhances the interaction of asphaltene molecules, making them more inclined to form face-to-face ordered stacking, and the hetero atom effect is more significant in increase of viscosity.

## Conflicts of interest

The authors declare that they have no known competing financial interests or personal relationships that could have appeared to influence the work reported in this paper.

## Supplementary Material

RA-014-D3RA06483B-s001
