# Supplementary material for: Molecular dynamics simulations of aggregation and viscosity properties of model asphaltene molecules containing a polycyclic hydrocarbon nucleus with toluene additive under shear interactions
Source: RSC Adv. 2024 Jan 15;14(4):2577–89. doi: 10.1039/d3ra06483b (PMC10788708; doi:10.1039/d3ra06483b)
Supplement: RA-014-D3RA06483B-s001 [file RA-014-D3RA06483B-s001.pdf]

Electronic Supplementary Material (ESI) for RSC Advances.
This journal is © The Royal Society of Chemistry 2023

Supporting Information

Molecular Dynamics Simulations of Aggregation and Viscosity Properties of Model Asphaltene Molecules Containing Polycyclic Hydrocarbon Nucleus with Toluene Additive under Shear Interactions

Baoliang Peng[1,*], Lingfang Yuan[2], Xianqiong Tang*[2], Yao Wang [3], Yingying Li[1], Weidong Liu[1], Yong Pei[2]

[1] *Research Institute of Petroleum Exploration & Development (RIPED), PetroChina, Key Laboratory of Oilfield Chemistry, CNPC, Beijing, People's Republic of China, 100083;*

[2] *Key Laboratory for Green Organic Synthesis and Application of Hunan Province, Key Laboratory of Environmentally Friendly Chemistry and Applications of Ministry of Education, Xiangtan University, Xiangtan, People's Republic of China, 411100;*

[3] *PetroChina Liaohe Oilfield Company, Panjin, People's Republic of China, 124010*

Table S1 Calculated dipole moment (in unit of Debye) of three types of asphaltene molecules. [a]Calculated by Gaussian 09 package; [b]Calculated based on PCFF force field parameters.

	PAHs-I	PAHs-L	PAHs-O	PAHs-T	PAHs-Y
Type-0	0.03[a] / 0.05[b]	1.37[a] / 0.30[b]	0.10[a] /0.02[b]	0.22[a] / 0.03[b]	0.10[a] / 0.17[b]
Type-1	2.94[a] / 2.55[b]	0.77[a] / 0.20[b]	1.53[a] /0.03[b]	4.17[a] / 0.02[b]	3.30[a] / 0.36[b]
Type-2	8.67[a] / 6.51[b]	11.22[a] / 3.46[b]	10.99[a] /1.77[b]	6.46[a] / 9.55[b]	7.25[a] / 8.06[b]

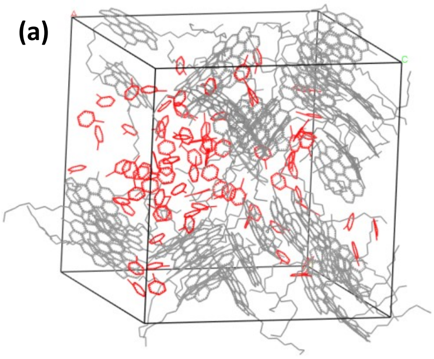

O1, 20Wt% toluene

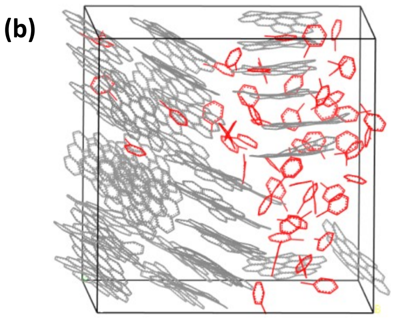

O0, 20Wt% toluene

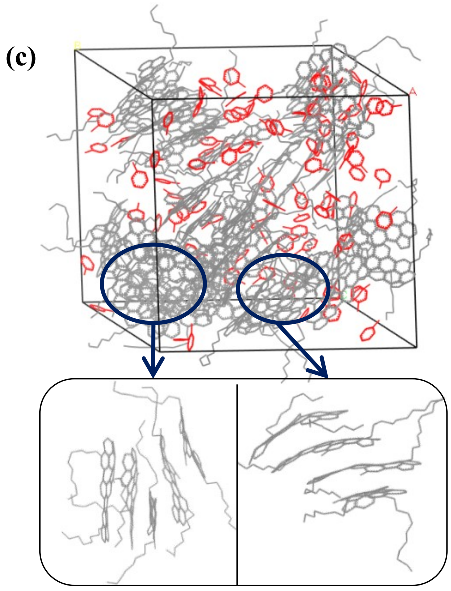

I1, 20Wt% toluene

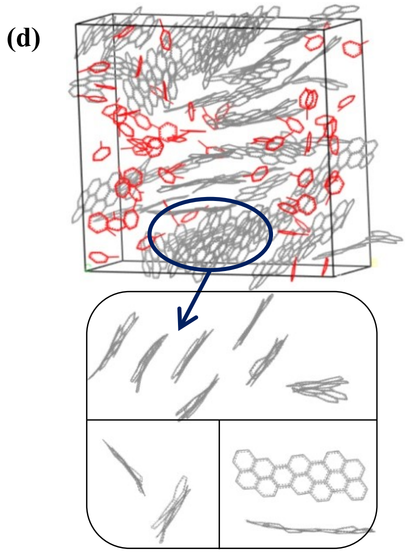

I0, 20Wt% toluene

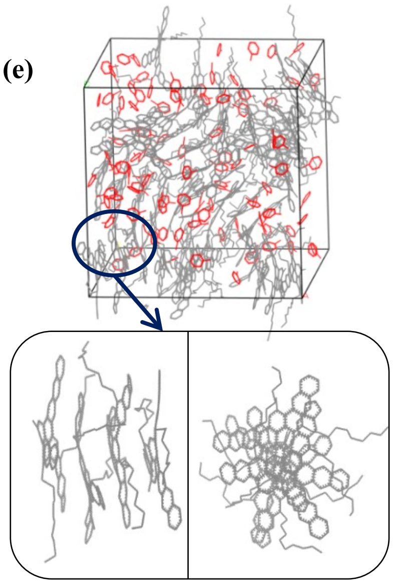

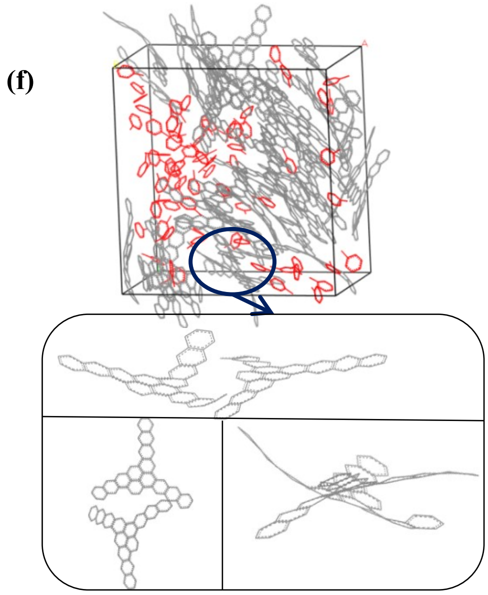

Y1, 20Wt% toluene

Y0, 20Wt% toluene

Figure S1 Snapshot structures of (a) PAHs-O1, (b) PAHs-O0, (c) PAHs-L1, (d) PAHs-L0, (e) PAHs-L1, and (y) PAHs-L0 type asphaltene molecular aggregates in the presence of 20 wt% toluene additive. Red lines shows the toluene molecules, grey lines represent the carbon atom skeletons. H atoms are omitted.

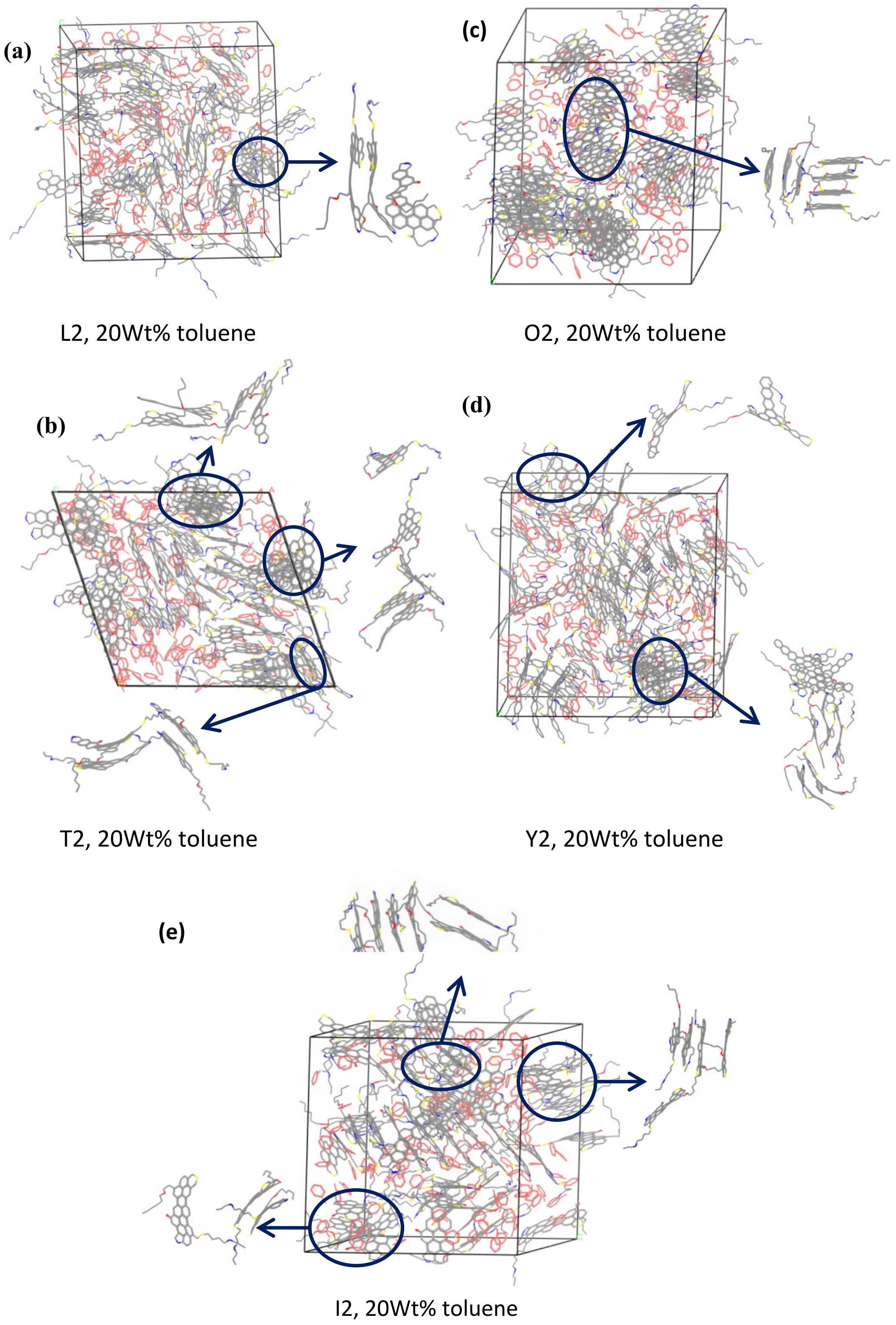

Figure S2 Snapshot structures of (a) PAHs-L2, (b) PAHs-O2, (c) PAHs-T2, (d) PAHs-Y2, and (e)

PAHs-I2 asphaltene molecular aggregates in the presence of 20 wt% toluene additive. Red lines shows the toluene molecules, grey lines represent the carbon atom skeletons. H atoms are omitted.
